# Skills, attitudes and uptake of evidence-based practice: a cross-sectional study of chiropractors in the Swedish Chiropractic Association

**DOI:** 10.1186/s12998-020-00359-w

**Published:** 2021-01-11

**Authors:** Matthew J. Leach, Per J. Palmgren, Oliver P. Thomson, Gary Fryer, Andreas Eklund, Stina Lilje, Jon Adams, Eva Skillgate, Tobias Sundberg

**Affiliations:** 1 National Centre for Naturopathic Medicine, Southern Cross University, East Lismore, NSW Australia; 2 Australian Research Centre in Complementary and Integrative Medicine, School of Public Health, Faculty of Health, University of Technology Sydney, Sydney, NSW Australia; 3 Department of Learning, Informatics, Management and Ethics, Karolinska Institutet, Stockholm, Sweden; 4 Research Centre, University College of Osteopathy, London, UK; 5 College of Health & Biomedicine, Victoria University, Melbourne, Victoria Australia; 6 Unit of Intervention and Implementation Research for Worker Health, Institute of Environmental Medicine, Karolinska Institutet, Stockholm, Sweden; 7 Musculoskeletal and Sports Injury Epidemiology Center, Department of Health Promotion Sciences, Sophiahemmet University, Stockholm, Sweden

**Keywords:** Evidence-based practice, Chiropractic, Health care surveys, Cross-sectional studies

## Abstract

**Background:**

Evidence-based practice (EBP) is integral to the delivery of high-quality health care. Chiropractic has been a licensed health profession in Sweden since 1989, but little is known of the uptake of EBP in this professional group. This study explored the self-reported skills, attitudes and uptake of EBP, and the enablers and barriers of EBP uptake, among licensed chiropractors in Sweden.

**Methods:**

Licensed chiropractors (*n* = 172) of the Swedish Chiropractic Association (Legitimerade Kiropraktorers Riksorganisation) were invited to participate in an anonymous online questionnaire, using the Evidence-Based Practice Attitude and Utilisation Survey (EBASE) in February 2019.

**Results:**

Fifty-six (33%) chiropractors completed the survey. Participants were predominantly male, aged 30–49 years, held a Master’s degree, and had received their highest qualification and practiced chiropractic for over a decade. Chiropractors rated their EBP skill-level mostly in the moderate to moderate-high range. The majority of chiropractors reported positive attitudes towards EBP, with most agreeing or strongly agreeing that EBP is necessary in the practice of chiropractic, and that EBP assists in making decisions about patient care. Chiropractors reported an average level of engagement in EBP activities. All participants indicated professional literature and research findings were useful in their day-to-day chiropractic practice. The main perceived enabler of EBP uptake was internet access in the workplace, whereas the main barrier to EBP uptake was lack of clinical evidence in chiropractic.

**Conclusions:**

Participating chiropractors of the Swedish Chiropractic Association were generally favourable of EBP, though only reported modest levels of EBP-related skills and engagement in EBP activities. Our findings suggest future studies investigating interventions focussed on improving chiropractors’ skills and uptake of EBP are warranted.

## Background

Contemporary health care requires practitioners to make clinical decisions that are patient-centred, collaborative, individualised for the patient, and informed by research evidence [1, 2]. Since the inception of evidence-based practice (EBP) – the incorporation of research evidence with a patient’s values and preferences and a practitioner’s clinical judgement and experience [3] – it has been emphasised that ‘evidence’ should be from the highest or ‘best’ quality research studies, namely results from systematic reviews and randomised controlled trials [4]. More recently, it has been acknowledged that the practice of EBP also needs to consider a range of research methodologies (qualitative and quantitative research), alongside a greater appreciation for the role of clinical judgement in the context of the patient’s individual life and circumstances [1].

Chiropractic can be defined as a health care profession focussed on diagnosing, treating and preventing mechanical disorders of the musculoskeletal system, predominantly relying on manual therapy interventions including spinal manipulation [5]. Recent chiropractic clinical guideline recommendations have expanded this scope of practice to include pain education, exercise and reassurance [6]. Not dissimilar to other health disciplines, chiropractic is beginning to shift away from a dependence on early theories and hypotheses to explain observed clinical improvements during chiropractic care, to embracing the concepts of modern scientific methodology and evidence [7].

In other physical and manual therapies, such as physiotherapy and osteopathy, there have been calls for these professions to incorporate EBP into their practice models and philosophies [8–11]. In chiropractic, similar calls have been made to accept and incorporate EBP [12, 13], with some claiming that chiropractic is at a crossroads, facing a choice between EBP and ideological dogma [14]. Congruent with this, the past decade has seen a gradual shift towards the EBP model within the chiropractic profession with the development of practice-based guidelines, EBP educational programmes and evidence-informed practice statements by regulatory/licencing authorities as well as national and international associations [12, 15]. It also has been recommended that Councils on Chiropractic Education, an international umbrella organisation of chiropractic accrediting bodies, further incorporate accepted mainstream healthcare standards, including evidence-based approaches, to accreditation standards, processes and chiropractic care [12, 16–18].

The Swedish National Board of Health and Welfare issues licences for health professions that meet the requirements of the state to practice health care [19]. Chiropractic has been a licensed health profession in Sweden since 1989 [20, 21]. To become a licensed chiropractor in Sweden the presumptive candidate needs to present a degree from a chiropractic educational institution acknowledged by the Swedish National Board of Health and Welfare [22]. This typically involves four academic years of theoretical and practical chiropractic studies. Chiropractors trained abroad also need to show adequate language skills in Swedish, Norwegian or Danish, and adequate professional skills by passing a theoretical and practical proficiency test, in order to apply for a license to practice chiropractic in Sweden [23–25]. In 2017, there were approximately 740 licensed chiropractors working in Sweden, of which 590 (80%) were working in health care where most were males (63%), aged between 45 and 49 years (15%), and working in the private sector (91%) [26]. The relative number of chiropractors working in the Swedish health care system increased with 20% from 2013 to 2017, with the Stockholm region boasting the highest number of chiropractors relative to the population (11 chiropractors per 100,000 inhabitants) in Sweden in 2017 [26]. Chiropractors in Sweden practice as portal of entry contacts for musculoskeletal conditions but are not typically authorised to operate their own diagnostic imaging equipment, provide specialist referrals or certify absence due to sickness. Although some regions may have limited local agreements, there is generally no public reimbursement from the Swedish government for patients receiving chiropractic care [27]. Chiropractors practising in Sweden are not required by law to be members of a professional association. Currently there is just one recognized association, Legitimerade Kiropraktorers Riksorganisation (the Swedish Chiropractic Association), that is an established member of the World Federation of Chiropractic, which is a member of the World Health Organization, and a member of the European Chiropractors’ Union [28]. The Swedish Chiropractic Association had 172 licensed chiropractor members as of February 2019 (Swedish Chiropractic Association personal communication 2019), thus representing approximately 23% (172/740) of all Swedish licensed working chiropractors and 29% (172/590) of all licensed chiropractors working in health care [26].

Previous studies exploring perceptions and uptake of EBP among chiropractors in the US, Canada and Australia have revealed positive attitudes towards EBP and satisfactory self-reported skills in EBP. However, these same studies also uncovered important barriers to EBP implementation, such as lack of time, perceived lack of evidence and research relevance, with many chiropractors reporting that they neither used clinical practice guidelines nor routinely applied evidence in clinical practice [29–33]. Despite the emerging body of work on EBP in chiropractic, it is uncertain if these findings apply to chiropractors in Sweden as no such studies have been conducted in this population. Addressing this knowledge gap will help informing the educational, research and regulatory needs of the Swedish chiropractic profession in terms of EBP.

The study presented in this paper was developed in direct response to this knowledge gap, with the aim to describe the self-reported skill-level, uptake and attitudes toward EBP, as well as the enablers and barriers to EBP uptake, among chiropractors in the Swedish Chiropractic Association. To address these aims, the study set out to answer the following research questions:

- What is the perceived level of skill in EBP among the chiropractors?
- What is the attitude toward EBP among the chiropractors?
- What is the level of engagement in EBP activities among the chiropractors?
- What do the chiropractors identify as enablers of, and barriers to EBP uptake?
- Are there any associations between EBP skill-level, use and attitude, and participant demographic factors?

## Methods

### Design

Cross-sectional survey.

### Sample and setting

The Swedish Chiropractic Association, representing 172 licensed chiropractors as of February 2019, was invited and agreed to participate in the survey. To achieve a response distribution of 50%, margin of error of 12% and confidence interval of 95% for any individual item in the survey, we needed to survey at least 49 of the 172 licensed chiropractors of the Swedish Chiropractic Association.

### Measurement

The Evidence-Based practice Attitude and utilization SurvEy (EBASE) is a 83-item, multidimensional instrument designed to gather information on the following constructs: level of skill in EBP, attitude toward EBP, uptake of EBP, training and education in EBP, enablers of EBP uptake and barriers to the uptake of EBP. A series of demographic items is also integrated into EBASE (e.g. age, sex, highest qualification, clinical setting, geographical region). EBASE has been administered to more than 8 distinct health professions to date [29, 30, 32, 34–38], and has been shown through psychometric testing to have good construct and content validity, acceptable test-retest reliability and good internal consistency [39, 40]. A useful feature of EBASE is its ability to generate three subscores. These include a skill subscore (with values ranging from 13 [primarily low-level skill] to 65 [primarily high-level skill]), ‘attitude subscore’ (with values ranging from 8 [predominantly strongly disagree] to 40 [predominantly strongly agree]) and ‘use subscore’ (with values ranging from 0 [mainly infrequent use] to 24 [mainly frequent use]) [32].

To ensure EBASE was suitable to administer to a Swedish chiropractic population, we adapted the WHO process for translating instruments [41]: we translated the survey from English into Swedish, had an external translator contribute to backwards translation, and conducted cognitive interviewing with a survey developer. The survey was pilot tested on a convenience sample of 10 respondents from various professions including chiropractic, physical therapy, naprapathy, osteopathy, social work, and administration. As the EBASE was initially developed for Australian complementary medicine (CM) providers, some survey items were modified to ensure they were suitable for chiropractors in Sweden. This included replacing the term ‘CM’ with ‘chiropractic’, ‘Australian States’ with ‘Counties of Sweden’, and interventions usually provided in an initial CM consultation to those more pertinent to Swedish chiropractic practice. These changes did not impact the meaning of the items.

### Recruitment and data collection

The Swedish Chiropractic Association emailed a web link to the anonymous EBASE survey to their licensed chiropractor members (*n* = 172) in February 2019. The survey was open for 2 months (February to April 2019) during which the licensed chiropractors received two reminders to participate at one and 3 weeks after the first invite. The survey was hosted online by SUNET Artologik, a secure web based survey system used in Swedish higher education [42].

### Data analysis

Survey data were coded in accordance with EBASE scoring guidelines [32]. Analyses were performed using IBM® SPSS® Statistics 25.0 (Armonk, New York, IBM Corp). We reported any skipped items as missing data. Associations between ordinal-level variables (e.g. hours/week in clinical practice, years since receiving highest qualification) were analysed using Kendall’s Tau correlation coefficient (Ƭ). Relationships between nominal-level variables (e.g. clinical setting, sex) were tested using Cramer’s V. We interpreted coefficients as follows: weak correlation (0.10–0.29), moderate correlation (0.30–0.49), and strong correlation (0.50–1.00) [43]. Medians and the interquartile range were used to report non-normally distributed data, while measures of central tendency and variability were used to report normally distributed data. Categorical data were reported as frequency distributions and percentages.

## Results

### Response rate and demographics

A total of 56 chiropractors completed the survey, resulting in a 33% (56/172) response rate.

Participants were predominantly male (57.1%), aged between 30 and 49 years (59.0%), and held a Master’s degree qualification or higher (71.4%). Most participants had received their highest qualification 11 or more years ago (71.4%) and had practiced in the field of chiropractic for ≥11 years (76.8%). Almost two-thirds (62.5%) of participants reported working 31–45 h per week in clinical practice. More than one-third (37.5%) of chiropractors participated in research, with only 3.6% reporting teaching in higher education. Most participants practiced in an urban location (76.8%) with a group of conventional providers (26.8%) or a group of conventional and CM providers (23.2%) (Table 1). These participants were largely located in the counties of Stockholm (33.9%) and Skåne (19.6%). Treatments provided by most participants in the first chiropractic consultation were joint manipulation (92.9%), triggerpoint therapy (83.9%) and home exercise and ADL advice/instruction (78.6%).

**Table 1:** Demographic characteristics of sample (*n* = 56)

Characteristic	Frequency
**Age**, n (%)	
20–29 years	3 (5.4)
30–39 years	17 (30.4)
40–49 years	16 (28.6)
50–59 years	13 (23.2)
60+ years	7 (12.5)
**Sex**, n (%)	
Male	32 (57.1)
Female	22 (39.3)
Do not wish to state	2 (3.6)
**Highest qualification**, n (%)	
Vocational Degree/Diploma	2 (3.6)
University or College Certificate/Diploma	2 (3.6)
Bachelor degree	4 (7.1)
Master’s degree (2 years)	35 (62.5)
PhD/Doctorate	5 (8.9)
Other	6 (10.7)
Missing	2 (3.6)
**Years since receiving highest qualification**, n (%)	
0 years	0 (0.0)
1–5 years	7 (12.5)
6–10 years	8 (14.3)
11–15 years	11 (19.6)
16+ years	29 (51.8)
Missing	1 (1.8)
**Years practiced in the field of chiropractic**, n (%)	
0 years	0 (0.0)
1–5 years	7 (12.5)
6–10 years	6 (10.7)
11–15 years	10 (17.9)
16+ years	33 (58.9)
**Hours per week in clinical (chiropractic) practice**, n (%)	
0 h	1 (1.8)
1–15 h	1 (1.8)
16–30 h	18 (32.1)
31–45 h	35 (62.5)
46+ hours	1 (1.8)
**Hours per week participating in research**, n (%)	
0 h	35 (62.5)
1–15 h	17 (30.4)
16–30 h	1 (1.8)
31–45 h	3 (5.4)
46+ hours	0 (0.0)
**Hours per week teaching higher education**, n (%)	
0 h	53 (94.6)
1–15 h	2 (3.6)
16–30 h	0 (0.0)
31–45 h	0 (0.0)
46+ hours	0 (0.0)
Missing	1 (1.8)
**Treatments/management typically provided in initial chiropractic consultation**, n (%)	
Joint manipulation (e.g. HVLA)	52 (92.9)
Triggerpoint therapy	47 (83.9)
Home exercise and ADL advice or instruction	44 (78.6)
Exercise and physical activity advice or instruction	43 (76.8)
Ergonomic advice or instruction	41 (73.2)
Health/lifestyle advice or instruction	37 (66.1)
Joint mobilisation	33 (58.9)
Referral to other healthcare provider	31 (55.4)
Physical exercise / rehabilitation training	27 (48.2)
Stretching	27 (48.2)
Non-prescription pharmaceutical advice or instruction	26 (46.4)
Massage/soft-tissue mobilization	24 (42.9)
Traction	23 (41.1)
Taping	20 (35.7)
Referral to other health service	16 (28.6)
Dietary advice or instruction	12 (21.4)
Heat/cold treatment	11 (19.6)
Other	10 (17.9)
Nutritional supplementation advice	9 (16.1)
Acupuncture	6 (10.7)
Laser therapy	4 (7.1)
Ultrasound	1 (1.8)
TENS	0 (0.0)
**Clinical setting in which chiropractic is predominantly practiced**, n (%)	
With a group of conventional providers	15 (26.8)
With CM & conventional providers	13 (23.2)
With a group of chiropractors	11 (19.6)
Solo practice	11 (19.6)
Other	3 (5.4)
With a group of CM providers	2 (3.6)
Within an educational institution (e.g. university)	1 (1.8)
**County regions of Sweden**, n (%)	
Stockholm county region	19 (33.9)
Skåne county region	11 (19.6)
Other county regions	26 (46.4)
Missing	1 (1.8)
**Geographical region**, n (%)	
City (Central business district)	43 (76.8)
Suburbs	11 (19.6)
Rural/remote region	1 (1.8)
Missing	1 (1.8)

*ADL* Activities of daily living, *CM* Complementary medicine, *HVLA* high-velocity low amplitude, *TENS* Transcutaneous electrical nerve stimulation

### Skills in EBP

Thirteen EBP-related skills were presented in EBASE. The highest levels of perceived skill (i.e. those reported mostly in the moderate-high to high range) related to the identification of precise clinical questions (73.2%), and the areas of evidence acquisition (i.e. locating professional literature [58.9%] and online database searching [55.4%]), and evidence application (i.e. applying research evidence to patient cases [69.7%], and using findings from clinical research [62.5%] and systematic reviews [53.6%]) (Table 2). Skills largely reported in the low to moderate range were conducting clinical research (71.4%) and conducting systematic reviews (76.8%).

**Table 2:** Respondent self-reported skills in evidence-based practice (*n* = 56)

	1 Low n (%)	2 Low-moderate n (%)	3 Moderate n (%)	4 Moderate-high n (%)	5 High n (%)	Median (IQR)
**Identifying precise clinical questions**	1 (1.8)	2 (3.6)	12 (21.4)	**30 (53.6)**	11 (19.6)	4 (3,4)
**Identifying knowledge gaps in practice**	1 (1.8)	1 (1.8)	**23 (41.1)**	19 (33.9)	12 (21.4)	4 (3,4)
**Locating professional literature**	1 (1.8)	8 (14.3)	14 (25.0)	**20 (35.7)**	13 (23.2)	4 (3,4)
**Online database searching**	4 (7.1)	8 (14.3)	13 (23.2)	**17 (30.4)**	14 (25.0)	4 (3,5)
**Retrieving evidence**	2 (3.6)	15 (26.8)	12 (21.4)	**16 (28.6)**	11 (19.6)	3 (2,4)
**Critical appraisal of evidence**	3 (5.4)	9 (16.1)	**17 (30.4)**	16 (28.6)	11 (19.6)	3 (3,4)
**Synthesis of research evidence**	3 (5.4)	13 (23.2)	**16 (28.6)**	14 (25.0)	10 (17.9)	3 (2,4)
**Applying research evidence to patient cases**	0 (0.0)	2 (3.6)	15 (26.8)	**31 (55.4)**	8 (14.3)	4 (3,4)
**Sharing evidence with colleagues**	6 (10.7)	11 (19.6)	11 (19.6)	**18 (32.1)**	10 (17.9)	4 (2,4)
**Using findings from systematic reviews**	5 (8.9)	6 (10.7)	15 (26.8)	**16 (28.6)**	14 (25.0)	4 (3,5)
**Using findings from clinical research**	1 (1.8)	5 (8.9)	15 (26.8)	**24 (42.9)**	11 (19.6)	4 (3,4)
**Conducting clinical research**	10 (17.9)	12 (21.4)	**18 (32.1)**	10 (17.9)	6 (10.7)	3 (2,4)
**Conducting systematic reviews**	**15 (26.8)**	13 (23.2)	**15 (26.8)**	9 (16.1)	4 (7.1)	3 (1,3)

*IQR* Interquartile range; main response in bold

The median skill subscore was 44.5 (IQR 35,51; range 21–64). This reflected a perceived skill level mostly in the moderate to moderate-high range, as defined by an overall value ranging between 39.1 and 51.9. A moderate positive correlation was observed between the skill subscore (categorised by quartiles) and highest qualification (Ƭ = 0.473, *p* < 0.001), hours per week participating in research (Ƭ = 0.497, *p* < 0.001) and practice setting (V = 0.482, *p* = 0.003; with higher skill levels observed among those working with conventional healthcare providers). There was a moderate negative correlation between the skill subscore and years since receiving the highest qualification (Ƭ = -0.325, *p* = 0.003). No statistically significant associations were found between the skill subscore and other demographic factors.

### Attitude toward EBP

Most participants were favourable of EBP, with 82.2–100.0% agreeing or strongly agreeing to 7 of the 10 attitudinal statements (Table 3). More than half (51.8%) of participants were uncertain, disagreed or strongly disagreed that EBP takes into account a patient’s preference for treatment. The majority of participants disagreed or strongly disagreed that there was a lack of evidence from clinical trials to support most of the treatments used in their practice (69.7%), and that the adoption of EBP places an unreasonable demand on their practice (80.4%).

**Table 3:** Respondent attitudes toward evidence-based practice (*n* = 56)

	1 Strongly Disagree n (%)	2 Disagree n (%)	3 Neutral n (%)	4 Agree n (%)	5 Strongly Agree n (%)	Median (IQR)
**EBP is necessary in the practice of chiropractic**	0 (0.0)	0 (0.0)	1 (1.8)	15 (26.8)	**40 (71.4)**	5 (4,5)
**EBP improves the quality of my patient’s care**	0 (0.0)	0 (0.0)	4 (7.1)	18 (32.1)	**34 (60.7)**	5 (4,5)
**EBP assists me in making decisions about patient care**	0 (0.0)	0 (0.0)	1 (1.8)	23 (41.1)	**32 (57.1)**	5 (4,5)
**I am interested in learning or improving the skills necessary to incorporate EBP into my practice**	0 (0.0)	0 (0.0)	6 (10.7)	18 (32.1)	**32 (57.1)**	5 (4,5)
**Professional literature (i.e. journals & textbooks) and research findings are useful in my day-to-day practice**	0 (0.0)	0 (0.0)	0 (0.0)	25 (44.6)	**31 (55.4)**	5 (4,5)
**Prioritizing EBP within chiropractic practice is fundamental to the advancement of the profession**	0 (0.0)	1 (1.8)	6 (10.7)	16 (28.6)	**33 (58.9)**	5 (4,5)
**EBP takes into account my clinical experience when making clinical decisions**	0 (0.0)	3 (5.4)	7 (12.5)	22 (39.3)	**24 (42.9)**	4 (4,5)
**EBP takes into account a patient’s preference for treatment**	4 (7.1)	8 (14.3)	**17 (30.4)**	11 (19.6)	16 (28.6)	3 (3,5)
**There is a lack of evidence from clinical trials to support most of the treatments I use in my practice**	8 (14.3)	**31 (55.4)**	9 (16.1)	7 (12.5)	1 (1.8)	2 (2,3)
**The adoption of EBP places an unreasonable demand on my practice**	22 (39.3)	**23 (41.1)**	7 (12.5)	4 (7.1)	0 (0.0)	2 (1,2)

*EBP* Evidence-based practice, *IQR* Interquartile range; main response in bold

The median attitude subscore (32.5, IQR 30,35; range 26–38) was indicative of mostly agree to strongly agree responses, as defined by an overall score ranging between 32.0 and 40.0. There was a weak positive correlation between the attitude subscore (categorised by quartiles) and highest qualification (Ƭ = 0.261, *p* = 0.029), but not with any other demographic factors.

### Uptake of EBP

Most participants engaged in EBP-related activities (excluding lay literature) at least once in the past month (75.0–92.9%), typically between 1 and 5 times (37.5–60.7%), but generally no more than 10 times (62.4–82.2%) (Table 4). The most frequently reported activity was using online search engines to search for practice-related literature or research (49.9% engaged in this activity ≥6 times in the preceding month). The activity practiced least frequently was the use of lay literature (i.e. magazines, layperson/self-help books and non-government/non-education institution websites) to assist clinical decision-making, with 76.8% engaging in this activity ≤5 times in the past month.

**Table 4:** Respondent use of evidence-based practice (i.e. number of times each activity was undertaken over the last month) (*n* = 56)

	0 0 times n (%)	1 1–5 times n (%)	2 6–10 times n (%)	3 11–15 times n (%)	4 16+ times n (%)	Median (IQR)
**I have used an online search engine to search for practice related literature or research**	7 (12.5)	**21 (37.5)**	12 (21.4)	4 (7.1)	12 (21.4)	2 (1,3)
**I have used professional literature or research findings to assist my clinical decision-making**	4 (7.1)	**25 (44.6)**	6 (10.7)	4 (7.1)	17 (30.4)	1 (1,4)
**I have read/reviewed clinical research findings related to my practice**	6 (10.7)	**29 (51.8)**	7 (12.5)	5 (8.9)	9 (16.1)	1 (1,3)
**I have read/reviewed professional literature (i.e. professional journals & textbooks) related to my practice**	8 (14.3)	**27 (48.2)**	9 (16.1)	3 (5.4)	9 (16.1)	1 (1,2)
**I have consulted a colleague or industry expert to assist my clinical decision-making**	9 (16.1)	**29 (51.8)**	8 (14.3)	1 (1.8)	9 (16.1)	1 (1,2)
**I have used professional literature or research findings to change my clinical practice**	6 (10.7)	**34 (60.7)**	4 (7.1)	5 (8.9)	7 (12.5)	1 (1,2)
**I have used an online database to search for practice related literature or research**	14 (25.0)	**23 (41.1)**	7 (12.5)	2 (3.6)	10 (17.9)	1 (0,2)
**I have referred to magazines, layperson / self-help books, or non-government/non-education institution websites to assist my clinical decision-making**	20 (35.7)	**23 (41.1)**	4 (7.1)	4 (7.1)	5 (8.9)	1 (0,1)

*IQR* Interquartile range; main response in bold

A variety of information sources were used by participants to inform their decision-making (Table 5). Almost one-half (48.2%) of participants used published clinical evidence, traditional knowledge and clinical practice guidelines a lot or always. Experimental/laboratory evidence was used the least, with 50% of participants never using this information source.

**Table 5:** Information sources used by respondents to inform their clinical decision-making (*n* = 56)

Information source	Never used n (%)	Used a little n (%)	Used to a moderate extent n (%)	Used a lot n (%)	Always used n (%)	Missing n (%)	Median (IQR)
**Published clinical evidence**	0 (0.0)	6 (10.7)	21 (37.5)	**22 (39.3)**	5 (8.9)	2 (3.6)	4 (3,4)
**Traditional knowledge**	0 (0.0)	5 (8.9)	**23 (41.1)**	16 (28.6)	11 (19.6)	1 (1.8)	3 (3,4)
**Clinical practice guidelines**	3 (5.4)	8 (14.3)	17 (30.4)	**21 (37.5)**	6 (10.7)	1 (1.8)	3 (3,4)
**Personal preference**	2 (3.6)	10 (17.9)	**21 (37.5)**	20 (35.7)	3 (5.4)	0 (0.0)	3 (3,4)
**Patient preference**	1 (1.8)	13 (23.2)	17 (30.4)	**19 (33.9)**	5 (8.9)	1 (1.8)	3 (2,4)
**Personal intuition**	7 (12.5)	10 (17.9)	**18 (32.1)**	**18 (32.1)**	3 (5.4)	0 (0.0)	3 (2,4)
**Fellow practitioners or experts**	2 (3.6)	14 (25.0)	**24 (42.9)**	15 (26.8)	1 (1.8)	0 (0.0)	3 (2,4)
**Textbooks**	5 (8.9)	11 (19.6)	**27 (48.2)**	11 (19.6)	2 (3.6)	0 (0.0)	3 (2,3)
**Trial and error**	2 (3.6)	**30 (53.6)**	19 (33.9)	5 (8.9)	0 (0.0)	0 (0.0)	2 (2,3)
**Experimental/laboratory evidence**	**28 (50.0)**	14 (25.0)	8 (14.3)	3 (5.4)	0 (0.0)	3 (5.4)	1 (1,2)

*IQR* Interquartile range; main response in bold

Nearly one-half (46.4%) of participants indicated that a large proportion (76–99%) of their practice was based on evidence from clinical research, with 37.5% reporting a moderate proportion (51–75%), 8.9% a small proportion (26–50%) and 7.1% a very small proportion (1–25%). No participants reported their practice as being 100% based on clinical research evidence.

The median use subscore (8, IQR 6,13; range 1–24) reflected an average level of engagement in EBP activities between 1 and 10 times in the preceding month (defined as an overall score of 6.1–12.0). A moderate positive correlation was found between the use subscore (categorised by quartiles) and gender (V = 0.369, *p* = 0.019; with higher use observed among females) and hours per week participating in research (Ƭ = 0.300, *p* = 0.001). There were no statistically significant associations between the skill subscore and other demographic factors.

### Training in EBP

Participants had completed training in the areas of evidence-based practice (100%), the application of research evidence to clinical practice (96.4%), critical analysis/critical thinking (92.9%), conducting clinical research (87.5%) and conducting systematic reviews and meta-analyses (83.9%). In most cases (50.0–60.7%), the training was undertaken as a component/course/module of a multi-year undergraduate program.

### Enablers of and barriers to EBP uptake

Most (50.0–91.1%) participants believed that 9 of the 10 listed factors were ‘very useful’ enablers of EBP uptake. The factors favoured the most were: access to the internet in the workplace (91.1% reported this factor as ‘very useful’), access to free online databases in the workplace (73.2%), access to critical reviews of research evidence relevant to chiropractic (67.9%), access to critically appraised topics relevant to chiropractic (64.3%) and ability to download full-text journal articles (60.7%). The factor favoured the least was access to online tools to assist with conducting critical appraisals of multiple research papers, with 55.4% of respondents identifying this factor as not/slightly/moderately useful in improving EBP uptake.

The majority (60.7–92.9%) of participants indicated that all 13 potential barriers listed in EBASE were either not a barrier or only a minor barrier to EBP uptake. Those factors considered by most to not be a barrier to EBP uptake were: lack of colleague support for EBP (75.0%), lack of relevance to chiropractic practice (67.9%), lack of resources (67.9%), lack of interest in EBP (64.3%), lack of industry support for EBP (62.5%) and lack of incentive to participate in EBP (58.9%). Factors largely reported as minor or moderate barriers to EBP uptake included: lack of clinical evidence in chiropractic (78.5%), insufficient skills to critically appraise the literature (62.5%), lack of time (59.0%), insufficient skills for interpreting research (59.0%), insufficient skills to apply research findings to practice (58.9%), insufficient skills for locating research (55.4%) and patient preference for a particular treatment (53.5%).

## Discussion

The findings of this study provide insights into the self-reported skills, attitudes, and uptake of EBP among Swedish chiropractors who are members of the Swedish Chiropractic Association. Participating chiropractors reported a moderate to moderate-high level of perceived skill in EBP, an overall positive attitude towards EBP, and an average level of engagement in EBP activities. Workplace internet access was the most favoured enabler of EBP uptake, whereas a lack of clinical evidence in chiropractic was considered the main barrier for such uptake.

### Skills in EBP

Participating chiropractors reported a relatively high level of perceived skill in performing several aspects of the EBP process; in particular, asking precise clinical questions, acquiring evidence and applying evidence. By contrast, chiropractors were less confident regarding their level of skill in conducting clinical research and systematic reviews. This is consistent with findings from studies involving other health professions [35–37, 44].

It has been suggested that clinical health care providers are “consumers” rather than “producers” of scientific knowledge [45]; thus, clinicians should not be expected to learn how to conduct high-level clinical research, but they must possess the skills of retrieving, assessing, and using research evidence in clinical practice [46]. Considering that, it was encouraging from an EBP perspective to see that almost one-half of participating chiropractors used clinical practice guidelines and published clinical evidence either a lot or always to inform their clinical decision-making. However, two-thirds of participants did report using personal intuition to varying degrees to inform their clinical decision-making. While the current study does not provide insights into the potential implications of using personal intuition as an information source to guide chiropractors’ clinical decision-making, this is one important topic for future enquiry.

### Attitude toward EBP

Attitudes toward EBP were generally positive among participating chiropractors. This corroborates the favourable perceptions of EBP reported among Canadian and Australian chiropractors [30, 33], as well as other healthcare professions, including nurses, physicians, physiotherapists, osteopaths and occupational therapists [36, 38, 47–50]. The vast majority of our study participants agreed or strongly agreed that EBP aids clinical decision making, improves the quality of patient care, and is necessary in chiropractic practice. These findings suggest that participating chiropractors may be aware of some of the many benefits of EBP to the chiropractic profession and to patient outcomes.

Our study revealed a positive association between the participants’ attitude toward EBP and their highest qualification. This concurs with previous study findings, suggesting that holding a higher educational qualification, such as a Master’s degree, may contribute to a better understanding of evidence-based practice, and in turn, lead to a more favourable attitude towards EBP [51–53]. Thus, it is possible that the large proportion of participating chiropractors with Master’s degrees in our study has shifted attitudes in favour of EBP. If this is indeed the case, a strategic measure to foster future EBP integration into chiropractic practise may be to support chiropractors to achieve higher educational qualifications.

### Uptake, enablers of and barriers to EBP

Participating Swedish chiropractors reported a low to moderate level of engagement in EBP activities. This is consistent with the level of EBP uptake observed in other health professions [30, 35, 36, 38]. An important enabler of EBP uptake among participants was internet access at work, possibly because this infrastructure is necessary to facilitate online access to clinical guidelines and journal publications. Considering that electronic patient records and time scheduling are advised in current clinical practice standards, it is likely that most chiropractors would have access to the internet in their clinical practice settings. However, a perceived lack of time, coupled with insufficient skills to locate, interpret, critically appraise and apply research findings to practice, may in all likelihood hinder EBP uptake. These barriers also may have contributed to participants’ perceptions of a lack of clinical evidence in chiropractic. Previous studies of chiropractors have reported similar barriers and facilitators to EBP uptake [30, 32].

Chiropractic associations and unions may play a pivotal role in propelling policy and practice recommendations on the importance of EBP uptake, promoting advances in chiropractic research, and communicating evidence-based chiropractic to the profession and wider community, including government, patients and other health care providers. The European Chiropractors’ Union has recently attempted such communication at the European Parliament level [54]. Somewhat discouraging though is findings from recent research showing that chiropractors may be less willing to join a professional association than other health professions; this highlights the need for chiropractic organisations to be more active in promoting how they support and impact the development of the chiropractic workforce [55].

### Methodological considerations

There were several limitations to the current study. While the participant response rate of 33% was higher than that reported in previous chiropractic studies utilising EBASE [30, 32], it implicates that the findings of our study may not be generalizable to the entire population of licensed chiropractors in the Swedish Chiropractic Association. Similarly, as study participants were only sourced from one chiropractic association (the Swedish Chiropractic Association), the findings may not reflect the views of Swedish chiropractors that are members of another association or those that are not members of any association. This should be considered in the interpretation of the findings. Nonetheless, the demographic characteristics of the participating chiropractors were largely representative of the characteristics of the Swedish Chiropractic Association membership in terms of gender distribution, years in practice and practice locations (Swedish Chiropractic Association personal communication 2020). The demographics, in terms of age and sex, were also largely representative of licensed Swedish chiropractors working in health care 2017, where our sample had 39% women compared to 37%, and 29% were in the age range 40–49 years compared to 28% [26]. However, we suspect our sample may have comprised a greater proportion of chiropractors with a Master’s degree or higher compared to all Swedish chiropractors, albeit this needs to be confirmed in future studies. To ensure the views of all licensed Swedish chiropractors are represented in future research, it is recommended that studies look beyond recruiting chiropractors from professional associations alone. Statistics Sweden (Statistikmyndigheten SCB) and the registry of licensed health personnel at the National Board of Health and Welfare (Registret över legitimerad hälso- och sjukvårdspersonal (HOSP), Socialstyrelsen) represent two possible options through which researchers could engage with a recruitment base of all licensed chiropractors in Sweden.

We acknowledge that the EBASE survey is long, and that this could potentially contribute to survey fatigue. However, all 56 chiropractors that participated in the survey completed all survey items; as such, there was no indication of survey fatigue in this cohort of participants. Additional limitations of this study, such as self-selection bias, cannot be excluded due to the use of self-selection sampling. Meaning that it cannot be excluded that participants that had more interest in EBP may have been more likely to participate in the survey and vice versa. Potential implications of such selection bias, if present, could for example be that some participants’ EBP attitudes were more positive, and their levels of reported EBP engagement more frequent, than across all chiropractors of the Swedish Chiropractic Association. Also, as the Swedish version of EBASE was not psychometrically tested, it is uncertain if the Swedish version of the survey retained the same psychometric properties as the English (original) version of EBASE. These limitations should be taken into consideration when interpreting the findings of the study.

The EBASE survey did not include questions about the participants’ chiropractic institutional background nor their philosophical chiropractic orientation, which may be useful to include in future studies to inform additional perspectives of chiropractic in relation to EBP. Furthermore, it is likely that the barriers and facilitators to chiropractors’ uptake of EBP may be influenced by a range of personal values, professional norms, and clinical/working environments, e.g. multidisciplinary clinics where the use of EBP is established and expected, such as in medicine and physiotherapy, [56], and may lead to variation in the way chiropractors adopt EBP. The use of mixed methods and qualitative research designs [57] may provide a richer understanding of the factors and contexts which are local to the individual practitioner and which shape the utilisation of EBP in the chiropractic community more broadly.

Considering the current findings, it is suggested that important directions for future action should be to develop and evaluate interventions focussed on improving EBP skill level and EBP uptake among chiropractors. A better understanding of barriers and facilitators to EBP skill development and uptake is an important next step to overcoming the obstacles to EBP use in chiropractic. The use of observational data collection methods to explore how chiropractic attitudes toward EBP and perceptions of EBP skill level relate to real world chiropractic practice behaviour and decision-making, represent another key focus for future research.

## Conclusions

This is the first study to examine the skills, attitude and uptake of EBP, as well as the barriers and enablers of EBP uptake, among Swedish chiropractors who are members of the Swedish Chiropractic Association. Although participating chiropractors reported a generally positive attitude towards EBP, their level of engagement in EBP activities was modest. A number of perceived barriers to EBP uptake may have contributed to this modest level of engagement, including a lack of clinical evidence in chiropractic, lack of time and insufficient skills in locating, appraising and translating evidence into practice. Notwithstanding, a deeper exploration of the barriers and facilitators to EBP utilisation is warranted. The findings of such work would help inform innovative strategies aimed at improving EBP skill development and supporting EBP uptake among Swedish chiropractors.

## Data Availability

The dataset used and/or analysed during the current study is available, in aggregated format to maintain participant anonymity, from the corresponding author on reasonable request.
